# Lymphocyte Perturbations in Malawian Children with Severe and Uncomplicated Malaria

**DOI:** 10.1128/CVI.00564-15

**Published:** 2016-02-05

**Authors:** Wilson L. Mandala, Chisomo L. Msefula, Esther N. Gondwe, James J. Gilchrist, Stephen M. Graham, Paul Pensulo, Grace Mwimaniwa, Meraby Banda, Terrie E. Taylor, Elizabeth E. Molyneux, Mark T. Drayson, Steven A. Ward, Malcolm E. Molyneux, Calman A. MacLennan

**Affiliations:** a Malawi-Liverpool-Wellcome Trust Clinical Research Programme, College of Medicine, Blantyre, Malawi; b Department of Basic Medical Sciences, College of Medicine, University of Malawi, Blantyre, Malawi; c Liverpool School of Tropical Medicine, University of Liverpool, Liverpool, United Kingdom; d Department of Microbiology, College of Medicine, University of Malawi, Blantyre, Malawi; e Department of Paediatrics, University of Oxford, Oxford, United Kingdom; f Wellcome Trust Centre for Human Genetics, University of Oxford, Oxford, United Kingdom; g Department of Paediatrics, College of Medicine, University of Malawi, Blantyre, Malawi; h Centre for International Child Health, University of Melbourne Department of Paediatrics and Murdoch Children's Research Institute, Royal Children's Hospital, Melbourne, VIC, Australia; i Blantyre Malaria Project, Blantyre, Malawi; j Department of Internal Medicine, College of Osteopathic Medicine, Michigan State University, East Lansing, Michigan, USA; k School of Immunity and Infection, College of Medicine and Dental Sciences, University of Birmingham, Birmingham, United Kingdom; l Department of Medicine, College of Medicine, University of Malawi, Blantyre, Malawi; m Jenner Institute, Nuffield Department of Medicine, University of Oxford, Oxford, United Kingdom; n Wellcome Trust Sanger Institute, Cambridge, United Kingdom

## Abstract

Lymphocytes are implicated in immunity and pathogenesis of severe malaria. Since lymphocyte subsets vary with age, assessment of their contribution to different etiologies can be difficult. We immunophenotyped peripheral blood from Malawian children presenting with cerebral malaria, severe malarial anemia, and uncomplicated malaria (*n* = 113) and healthy aparasitemic children (*n* = 42) in Blantyre, Malawi, and investigated lymphocyte subset counts, activation, and memory status. Children with cerebral malaria were older than those with severe malarial anemia. We found panlymphopenia in children presenting with cerebral malaria (median lymphocyte count, 2,100/μl) and uncomplicated malaria (3,700/μl), which was corrected in convalescence and was absent in severe malarial anemia (5,950/μl). Median percentages of activated CD69^+^ NK (73%) and γδ T (60%) cells were higher in cerebral malaria than in other malaria types. Median ratios of memory to naive CD4^+^ lymphocytes were higher in cerebral malaria than in uncomplicated malaria and low in severe malarial anemia. The polarized lymphocyte subset profiles of different forms of severe malaria are independent of age. In conclusion, among Malawian children cerebral malaria is characterized by lymphocyte activation and increased memory cells, consistent with immune priming. In contrast, there are reduced memory cells and less activation in severe malaria anemia. Further studies are required to understand whether these immunological profiles indicate predisposition of some children to one or another form of severe malaria.

## INTRODUCTION

There are approximately 500 million clinical episodes of Plasmodium falciparum malaria each year, leading to around 0.65 million deaths worldwide, most of which occur in African children (1). Despite promising recent results from the RTS, S/AS01 phase 3 malaria vaccine trial in African children (2), no vaccine against malaria is currently available, and resistance of parasites to different treatments and of mosquitoes to insecticides is a growing problem (3). The development of new therapies and vaccines against malaria would be facilitated by an improved understanding of the pathogenesis of the disease (4). The publication of the full genome of P. falciparum in 2002 (5) has led to greater insights into the biology of the malaria parasite, its antigenic variability, and its genetic diversity (6). However, our understanding of the host-pathogen interaction, and particularly of the host immune response to malaria, is incomplete (7).

Immunity to malaria involves various mechanisms. Antibodies that develop through exposure to P. falciparum (8) play a role, and the involvement of different lymphocyte subsets has been implicated in both protection against and pathogenesis of malaria (9, 10). Many of the studies on which these findings have been based have involved the investigation of experimental malaria in animals or nonimmune volunteers. A number of clinical studies of lymphocyte populations have been conducted in African children (11–15). The results of these studies can be difficult to interpret, because large studies usually do not discriminate between different clinical manifestations of malaria and the lymphocyte subsets investigated are often limited. Small studies can suffer from insufficient numbers for statistical analysis and are often focused on one aspect of the immune response.

The two main forms of severe malaria, severe malarial anemia and cerebral malaria, occur in children with differing, although overlapping, age distributions, with cerebral malaria typically afflicting older children (16). In a study of lymphocyte subsets in healthy Malawians, we found a strong linear trend with age for the majority of parameters tested, with absolute lymphocyte counts decreasing with age (17). Hence, changes in lymphocyte subsets with malaria could reflect patient age rather than disease state. To better understand the pathogenesis of severe malaria and uncomplicated malaria in a population severely affected by malaria, we undertook a study of lymphocyte subsets among Malawian children. Clinical presentation was carefully discriminated between cerebral malaria, severe malarial anemia, and uncomplicated malaria. Patients' ages were recorded, and lymphocyte profiles were studied during acute illness and reexamined in convalescence. We investigated absolute and percent lymphocyte counts and lymphocyte activation and memory status.

## MATERIALS AND METHODS

### Study design and participants.

The study was conducted within the Malawi-Liverpool-Wellcome Trust Clinical Research Programme and Department of Pediatrics, College of Medicine, University of Malawi, and Blantyre Malaria Project. Participants were children admitted with malaria to Queen Elizabeth Central Hospital and medically well children selected to have approximately the same age range and distribution as study children with malaria attending surgical outpatient clinics at Queen Elizabeth Central Hospital and Beit Cure International Hospital, both in Blantyre. Children were enrolled during the rainy season (November to April). Following informed consent from the parent or guardian, each child was examined by a research nurse and clinical officer, baseline demographic data were recorded, and a peripheral blood sample was taken. Children were assessed for level of consciousness using the Blantyre Coma Score (BCS) on admission and at 2- to 4-hourly intervals during intensive clinical care. Over 40 children were prospectively enrolled into each of the four clinical groups defined by diagnoses of cerebral malaria, severe malarial anemia, or uncomplicated malaria or healthy controls.

Malaria was defined as a clinical syndrome without an apparent alternative cause, in the presence of a thick blood film positive for P. falciparum asexual parasites on microscopy. Children with cerebral malaria had a BCS of 2 or less at admission and 4 h later, while children in all other groups had a score of 5 at both times (Table 1). Children with severe malarial anemia had a blood hemoglobin concentration of 5 g/dl or less, and all other children had a hemoglobin concentration above this level. Children who tested positive for HIV infection were excluded from the study and referred to the antiretroviral therapy clinic.

**TABLE 1:** Eligibility criteria for recruitment into study groups

Group no.	Group name (abbreviation)	Result for criterion^*a*^:			
		MPS	BCS	Hb (g/dl)	HIV
1	Cerebral malaria (CM)	Positive	≤2	>5	−ve
2	Severe malarial anemia (SMA)	Positive	5	≤5	−ve
3	Uncomplicated malaria (UCM)	Positive	5	>5	−ve
4	Healthy controls	Negative	5	>5	−ve

a Abbreviations: MPS, malaria parasite slide; BCS, Blantyre coma score at presentation and 4 h later; Hb, hemoglobin; HIV, HIV status; −ve, uninfected.

### Investigations.

Investigations were performed on EDTA-anticoagulated blood on the day of venesection. HIV testing was performed using two rapid tests, Determine (Abbott Laboratories, Tokyo, Japan) and UniGold (Trinity Biotech, Dublin, Ireland). Discordant results in all children and positive results in children under 18 months were confirmed by PCR (18). Thick and thin films were prepared for determining the density of malaria parasitemia. A full blood count was performed on an HMX hematological analyzer (Beckman Coulter, California). For immunophenotyping, 25 μl blood was labeled with monoclonal antibodies in eight tubes according to the procedure described in Table S1 in the supplemental material. All antibodies were from Becton Dickinson (San Jose, CA) (see Table S2). Samples were processed, and data were acquired using a FACSCalibur flow cytometer and CellQuest software (Becton Dickinson) as previously described (17). The gating strategy used is shown in Fig. S1 in the supplemental material.

### Statistical analysis.

Medians and 10th and 90th percentiles were determined for absolute and percent lymphocyte subset concentrations in each clinical group at acute presentation and convalescence. Lymphocyte parameters were compared between control subjects and children with uncomplicated malaria, severe malarial anemia, and cerebral malaria using nonparametric Kruskal-Wallis tests. To assess whether age-related variations in lymphocyte counts confounded any observed associations, we also compared lymphocyte parameters by nonparametric analysis of covariance (ANCOVA) with age as a covariate in the model, using the sm package in R. Given the multiple lymphocyte parameters studied, Kruskal-Wallis and nonparametric ANCOVA *P* values were corrected for multiple comparisons using Benjamini-Hochberg adjustments, and adjusted *P* values of <0.05 were considered significant. For *post hoc* testing in cases of significant Kruskal-Wallis and nonparametric ANCOVA tests, Dunn's test pairwise comparisons were performed with Benjamini-Hochberg corrections for multiple comparisons. All statistical analyses were performed in R.

### Ethical approval.

The study was approved by the College of Medicine Research and Ethics Committee, University of Malawi, and the Ethics Committee of the Liverpool School of Tropical Medicine, United Kingdom. A parent or guardian of each participant provided informed written consent on the child's behalf.

## RESULTS

### Characteristics of study children.

Consent was given for 188 children aged 5 to 84 months to participate in the study. Blood samples from 33 children were excluded for the following reasons: HIV infection (*n* = 14), malaria parasites in the blood of control subjects (*n* = 14), BCS greater than 2 at 4 h postadmission in children with suspected cerebral malaria (*n* = 4), and hemoglobin below 5 g/dl in one child with cerebral malaria. The baseline characteristics of the remaining 155 children are presented in Table 2.

**TABLE 2:** Participant details^*a*^

Characteristic	Value for clinical group:			
	Control	Cerebral malaria	Severe malarial anemia	Uncomplicated malaria
No. of patients				
Total	42	29	30	54
Died during admission		4	1	0
Followed up in convalescence		18	21	34
Sex (male/female ratio)	29:13	10:19	19:11	38:16
Median age, mo (range)	20 (5–76)	30 (5–84)	23 (5–38)	27 (6–58)
Median no. of parasites/μl blood (range)	0	41,800 (900–517,000)	3,500 (20–296,000)	52,300 (460–768,000)
Median Blantyre coma score (range)	5	1 (0–2)	5	5
Median hemoglobin, g/dl (range)	11.2 (7.0–14.1)	7.7 (5.3–12.5)	3.9 (2.4–4.9)	9.3 (5.1–13.0)

a Subjects were children with cerebral malaria, severe malarial anemia, or uncomplicated malaria presenting to the Pediatric Accident and Emergency Clinic at Queen Elizabeth Central Hospital in Blantyre, Malawi. Control subjects were children admitted for elective surgical procedures who were medically well.

Median age was higher for children with cerebral malaria (30 months) than those with severe malarial anemia (23 months) (Mann-Whitney U test, *P* = 0.007), consistent with the reported age pattern of these two forms of malaria (16). Five children (four with cerebral malaria and one with severe malarial anemia) died during admission. Seventy-three out of 113 children with malaria returned for follow-up.

### Absolute cell counts indicate transient panlymphopenia in children with cerebral malaria.

**FIG 1:**
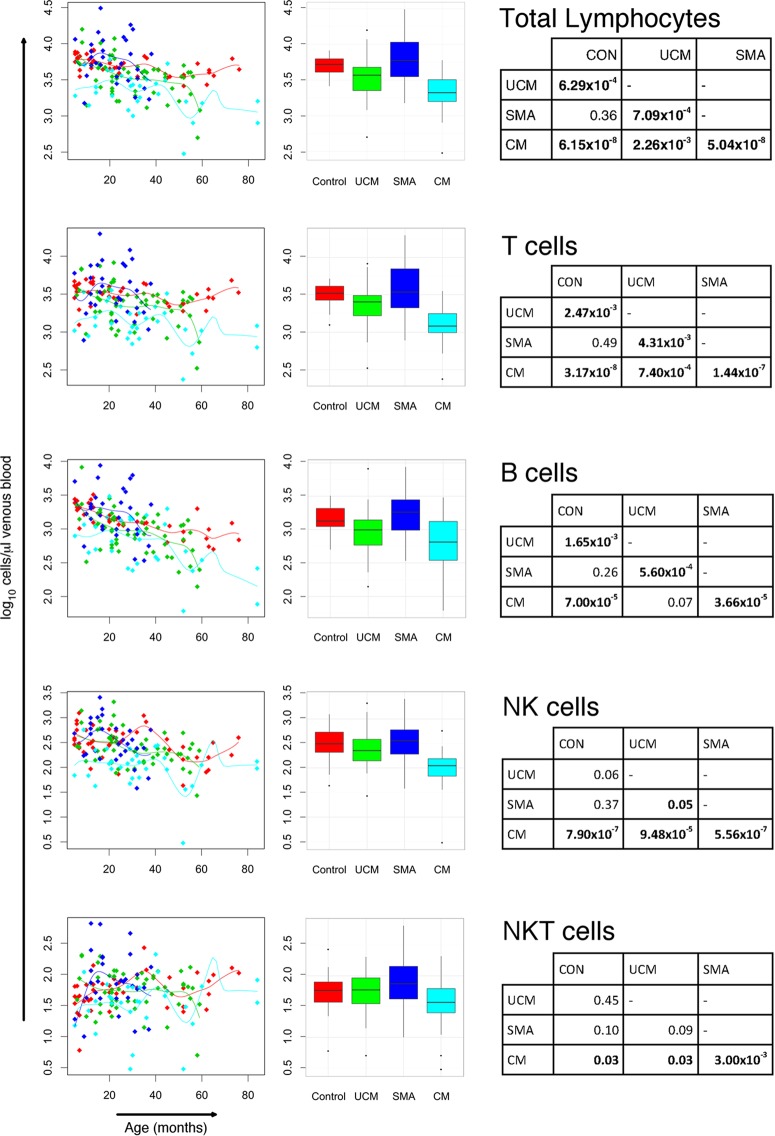
Lymphocyte subset counts in children with cerebral malaria (CM), severe malaria anemia (SMA), or uncomplicated malaria (UCM) and aparasitemic children. Total, T, B, NK, and NK T cell counts in peripheral blood at acute presentation with CM (turquoise), SMA (dark blue), or UCM (green) compared with healthy children (red). Counts shown for individual children against age with nonparametric regression line (left column) and box-and-whisker plots (middle column; boxes, interquartile ranges; whiskers, data ranges excluding outliers). Tables (right column) give Dunn's pairwise *post hoc* comparisons of groups where Kruskal-Wallis and nonparametric ANCOVA comparisons of data across groups gave *P* values of <0.05. All *P* values were adjusted with Benjamini-Hochberg corrections for multiple comparisons.

**FIG 2:**
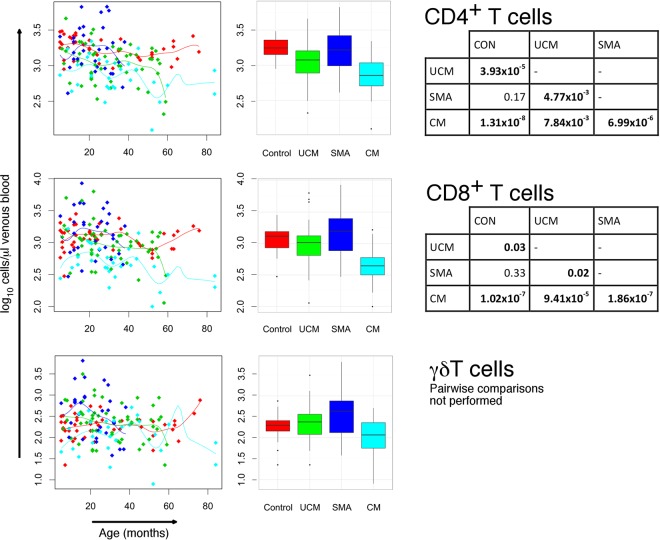
Absolute T lymphocyte subset counts (CD4^+^, CD8^+^, and γδ^+^ T cell) in children with cerebral malaria (CM), severe malaria anemia (SMA), or uncomplicated malaria (UCM) and aparasitemic children. CD4^+^, CD8^+^, and γδ^+^ T cell counts in peripheral blood at acute presentation with CM (turquoise), SMA (dark blue), or UCM (green) compared with healthy children (red). Counts shown for individual children against age with nonparametric regression line (left column) and box-and-whisker plots (middle column; boxes, interquartile ranges; whiskers, data ranges excluding outliers). Tables (right column) give Dunn's pairwise *post hoc* statistical comparisons of groups where Kruskal-Wallis and nonparametric ANCOVA comparisons of data across groups gave *P* values of <0.05. All *P* values were adjusted with Benjamini-Hochberg corrections for multiple comparisons.

Children with cerebral malaria had very low median total lymphocyte counts (2,100/μl) compared with children with uncomplicated malaria (3,700/μl) (*P* = 0.002), which, in turn, were lower (*P* < 0.001) than those of children with severe malarial anemia, who had a median value similar to that of the control group (5,950/μl and 5,250/μl, respectively) (Table 3; Fig. 1). The lymphopenia in these first two groups affected overall T and B lymphocyte subsets, as well as CD4^+^ and CD8^+^ T cell subsets (Table 3; Fig. 2). These changes are independent of age-related changes in lymphocyte counts. Despite the similarity of the median total lymphocyte counts in children with severe malarial anemia and in control children, the range of total lymphocyte counts among those with severe malarial anemia was broader. Median convalescent total lymphocyte counts were similar to counts of the control group for all malaria groups, indicating that the panlymphopenia of malaria is transient. Lymphocyte subset counts were normalized during convalescence following each form of malaria, and there were no significant differences between counts for each lymphocyte subset in each clinical group in convalescence and controls (Table 3).

**TABLE 3:** Absolute lymphocyte subset concentrations in venous blood from Malawian children with malaria

Lymphocyte subset	Median value^*a*^ (10th–90th percentile) for clinical group:							*P* value for^*b*^:			
	Control (*n* = 42)	Cerebral malaria		Severe malarial anemia		Uncomplicated malaria		Differences between acute groups and controls (Kruskal-Wallis, adjusted)	Acute group differences independent of age (nonparametric ANCOVA, adjusted)	Differences between Conv groups and controls (Kruskal-Wallis, adjusted)	Conv group differences independent of age (nonparametric ANCOVA, adjusted)
		Acute (*n* = 29)	Conv^*c*^ (*n* = 18)	Acute (*n* = 30)	Conv (*n* = 21)	Acute (*n* = 54)	Conv (*n* = 34)				
Total lymphocytes	5,250 (3,500–7,470)	2,100 (960–4,460)	4,750 (2,720–6,760)	5,950 (2,340–14,540)	5,300 (2,520–11,200)	3,700 (1,600–6,900)	5,200 (3,190–9,160)	8.2 × 10^−9^	5.6 × 10^−3^	0.90	0.37
T cells (CD3^+^)	3,317 (1,989–4,627)	1,208 (683–2,919)	3,414 (2,067–4,337)	3,446 (1,386–8,904)	3,558 (1,816–6,515)	2,543 (1,002–4,582)	3,671 (2,108–5,829)	1.7 × 10^−8^	5.6 × 10^−3^	0.90	0.23
CD4^+^ T cells (CD3^+^ CD4^+^)	1,763 (1,154–2,665)	717 (381–1,533)	1,812 (1,081–2,798)	1,651 (772–5,658)	1,873 (755–4,002)	1,189 (496–2,022)	1,678 (1,110–3,065)	1.4 × 10^−8^	0.011	0.95	0.60
CD8^+^ T cells (CD3^+^ CD8^+^)	1,304 (710–1,984)	442 (201–1,017)	1,268 (807–2,001)	1,581 (504–4,106)	1,550 (623–3,283)	1,029 (360–1,772)	1,197 (861–2,717)	6.1 × 10^−8^	0.045	0.90	0.23
γδ T cells (CD3^+^ γδ TCR^+^^*d*^)	198 (82–368)	116 (32–343)	391 (122–672)	432 (76–2,272)	296 (97–879)	239 (68–837)	290 (125–750)	1.5 × 10^−3^	0.19	0.074	0.52
B cells (CD19^+^)	1,342 (692–2,586)	651 (242–1,527)	761 (439–2,044)	1,836 (558–5,597)	1,223 (523–2,322)	990 (379–1,869)	1,070 (565–3,308)	4.3 × 10^−6^	0.021	0.44	0.60
NK cells (CD3^−^ CD56^+^)	310 (98–767)	111 (42–243)	266 (106–908)	358 (136–1,022)	484 (141–769)	225 (94–713)	317 (135–822)	3.8 × 10^−7^	0.037	0.81	0.60
NK T cells (CD3^+^ CD56^+^)	58 (25–114)	37 (10–87)	75 (30–138)	74 (18–266)	75 (29–167)	59 (22–115)	81 (28–170)	0.011	0.047	0.68	0.63

a Per microliter of blood.

b Kruskal-Wallis and nonparametric ANCOVA *P* values apply to groups of children with acute or convalescent malaria and controls and are adjusted with Benjamini-Hochberg corrections for multiple comparisons.

c Conv, convalescent.

d TCR, T cell receptor.

### Numbers of activated innate lymphocytes increase in malaria.

**FIG 3:**
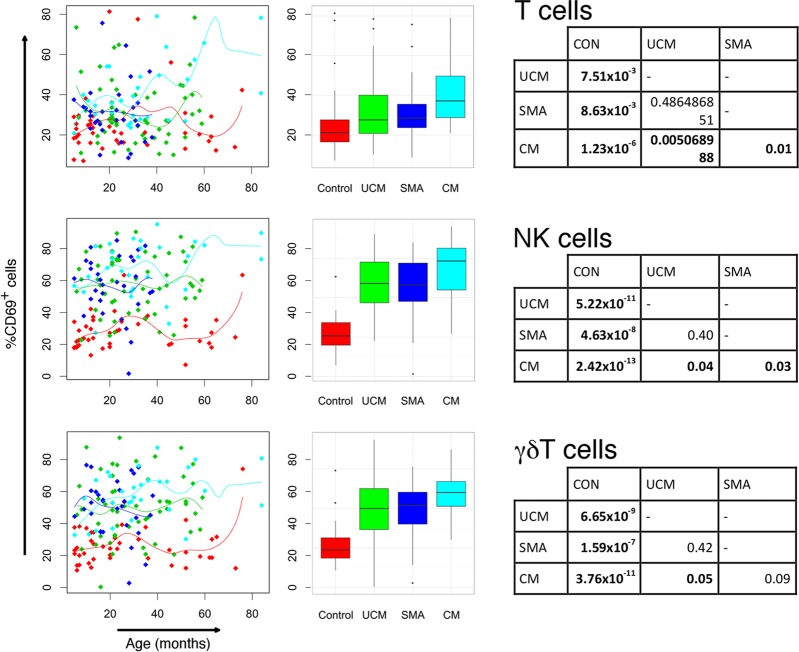
Percentages of activated lymphocytes in children with cerebral malaria (CM), severe malaria anemia (SMA), or uncomplicated malaria (UCM) and aparasitemic children. Percentages of T, NK, and γδ T lymphocytes expressing activation marker CD69 in peripheral blood at acute presentation with CM (turquoise), SMA (dark blue), or UCM (green) compared with healthy children (red). Counts shown for individual children against age with nonparametric regression line (left column) and box-and-whisker plots (middle column; boxes, interquartile ranges; whiskers, data ranges excluding outliers). Tables (right column) give Dunn's pairwise *post hoc* statistical comparisons of groups where Kruskal-Wallis and nonparametric ANCOVA comparisons of data across groups gave *P* values of <0.05. All *P* values were adjusted with Benjamini-Hochberg corrections for multiple comparisons.

All forms of malaria were associated with increased numbers of lymphocytes expressing the acute activation marker CD69 (Table 4; Fig. 3). This was particularly marked for innate lymphocytes, NK cells, and γδ T cells (for each acute malaria group compared to control group, *P* < 0.001) and most evident in children with cerebral malaria (median values of CD69^+^ cells, 73% for NK cells and 60% for γδ T cells). Median CD69-expressing lymphocytes were normalized in convalescence.

**TABLE 4:** Percentages of activated lymphocyte subset concentrations and ratios of memory to naive lymphocyte subsets in venous blood from Malawian children with malaria

Subset characteristic^*a*^	Median value (10th–90th percentile) for clinical group:							*P* value for^*b*^:			
	Control (*n* = 42)	Cerebral malaria		Severe malarial anemia		Uncomplicated malaria		Differences between acute groups and controls (Kruskal-Wallis, adjusted)	Acute group differences independent of age (nonparametric ANCOVA, adjusted)	Differences between Conv groups and controls (Kruskal-Wallis, adjusted)	Acute group differences independent of age (nonparametric ANCOVA, adjusted)
		Acute (*n* = 29)	Conv^*c*^ (*n* = 18)	Acute (*n* = 30)	Conv (*n* = 21)	Acute (*n* = 54)	Conv (*n* = 34)				
% CD69^+^ of subset											
T cells	21.08 (13.29–37.39)	37.09 (25.83–64.16)	27.13 (10.97–57.49)	28.46 (15.83–49.30)	24.10 (10.35–61.96)	27.55 (15.91–53.25)	25.26 (12.45–54.92)	1.4 × 10^−5^	9.2 × 10^−3^	0.83	0.60
NK cells	25.77 (18.06–37.81)	73.36 (48.63–85.23)	30.84 (18.07–48.38)	58.18 (25.08–79.45)	29.29 (17.79–48.62)	59.11 (34.56–78.34)	33.60 (15.85–49.90)	6.0 × 10^−14^	1.0 × 10^−4^	0.52	0.60
γδ T cells	23.83 (13.76–38.21)	60.13 (34.95–76.92)	24.13 (12.51–35.23)	51.94 (33.14–73.46)	25.62 (13.71–43.48)	50.00 (25.44–77.27)	25.76 (4.28–33.49)	1.2 × 10^−11^	2.0 × 10^−4^	0.95	0.61
Memory/naïve cell ratio of subset											
CD4^+^ T cells	0.40 (0.23–0.87)	0.85 (0.50–1.93)	0.55 (0.29–1.16)	0.40 (0.24–0.71)	0.47 (0.23–1.30)	0.62 (0.31–1.31)	0.49 (0.34–0.92)	5.7 × 10^−8^	5.6 × 10^−3^	0.47	0.60
CD8^+^ T cells	0.19 (0.11–0.37)	0.21 (0.09–0.45)	0.26 (0.08–0.45)	0.16 (0.07–0.42)	0.15 (0.09–0.53)	0.23 (0.11–0.56)	0.19 (0.10–0.48)	0.20	0.24	0.95	0.60
B cells	0.18 (0.06–0.54)	0.27 (0.10–0.45)	0.27 (0.08–0.59)	0.18 (0.08–0.33)	0.20 (0.09–0.40)	0.27 (0.10–0.44)	0.27 (0.11–0.47)	0.039	0.69	0.81	0.90

a Values are medians (10th and 90th percentiles) presented as percentages of each lymphocyte subset expressing the CD69 activation marker or memory-to-naive cell ratio of each subset according to CD45RO (memory T cell), CD45RA (naive T cell), and CD27 (memory B cell) expression.

b Kruskal-Wallis and nonparametric ANCOVA *P* values apply to groups of children with acute or convalescent malaria and controls and are adjusted with Benjamini-Hochberg corrections for multiple comparisons.

c Conv, convalescent.

### Cerebral malaria is associated with increased proportions of memory CD4^+^ T cells.

**FIG 4:**
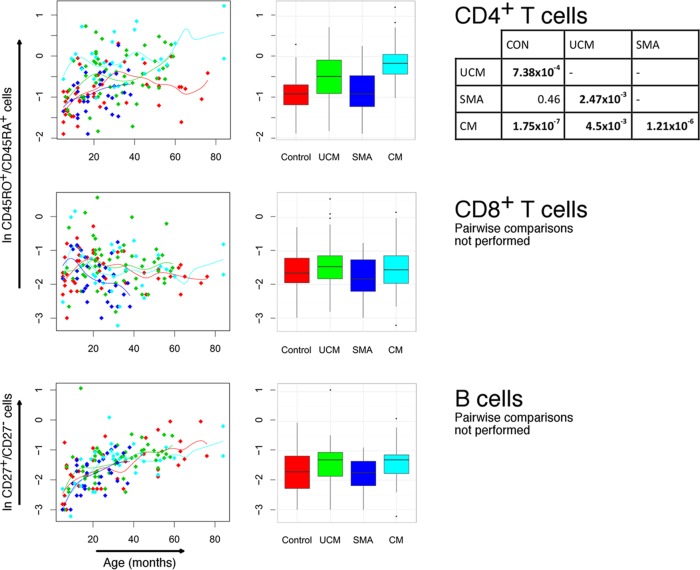
Memory/naive lymphocyte ratios in children with cerebral malaria (CM), severe malaria anemia (SMA), or uncomplicated malaria (UCM) and aparasitemic children. Ratios of CD45RO^+^ to CD45RA^+^ CD4^+^ and CD8^+^ T cells and CD27^+^ to CD27^−^ B cells in peripheral blood at acute presentation with CM (turquoise), SMA (dark blue), or UCM (green) compared with healthy children (red). Counts shown for individual children against age with nonparametric regression line (left column) and box-and-whisker plots (middle column; boxes, interquartile ranges; whiskers, data ranges excluding outliers). Tables (right column) give Dunn's pairwise *post hoc* statistical comparisons of groups where Kruskal-Wallis and nonparametric ANCOVA comparisons of data across groups gave *P* values of <0.05. All *P* values were adjusted with Benjamini-Hochberg corrections for multiple comparisons.

Using CD45RO and CD45RA to distinguish memory and naive T cells, respectively, we found a high ratio of memory to naive CD4^+^ lymphocytes in cerebral and uncomplicated malaria (median ratios, 0.85 and 0.62, respectively) compared with controls and children with severe malarial anemia (both 0.40) (cerebral malaria or uncomplicated malaria compared to severe malarial anemia or controls, *P* < 0.001 for all pairwise comparisons, except uncomplicated malaria and severe malarial anemia, where *P* was 0.002) (Table 4; Fig. 4). Despite children in cerebral and uncomplicated malaria groups being generally older than those with severe malarial anemia and controls, the differences between groups were apparent at all ages but did not persist in convalescence (Table 4). In contrast, there was no significant increase in memory to naive CD8^+^ T cells in any form of malaria compared with controls. The median ratios of memory to naive B cells (indicated by the presence or absence of CD27 expression) were not significantly different in children with malaria compared to controls.

## DISCUSSION

Perturbations of lymphocyte subsets in the context of different clinical forms of malaria have long been recognized (19, 20), but lymphocyte subset profiles also vary markedly with age, particularly in early childhood (17). Using careful malaria subtype case definitions and analysis of results with age, we have confirmed that the immunophenotype profiles of different forms of severe malaria are linked to the infection itself rather than patient age. Of the three clinical presentations of malaria examined, changes in lymphocyte distributions were most marked in children with cerebral malaria, where a panlymphopenia was observed affecting all three main lymphocyte subsets and T cell subsets. Normalization of lymphocyte numbers in convalescence indicates that the observed panlymphopenia is transient and is consistent with this not being a predisposing cause of cerebral malaria. The closely matching percent cell count distributions for cerebral malaria and healthy children indicate that the panlymphopenia is almost all attributable to changes in the total lymphocyte count.

The observation that panlymphopenia is most marked in cerebral malaria while absent in severe malaria anemia is somewhat counterintuitive, given the apparent role of T cell-dependent immunopathology in the pathogenesis of cerebral malaria, characterized by high levels of proinflammatory cytokines (9, 10). This apparent paradox may be partly explained by the fact that only 5% of recirculating lymphocytes are present in peripheral blood, with lymphocytes principally developing immune responses within secondary lymphoid compartments of the spleen and lymph nodes. Lymphopenia has previously been described in the context of clinical malaria (21, 22) and has been attributed to temporary sequestration of cells in secondary lymphoid tissue (13, 23) or to lymphocyte apoptosis (24, 25). While absolute lymphocyte counts were normalized in convalescence from cerebral and uncomplicated malaria, it is striking that this recovery is characterized by a reduction in the proportion of B cells, leading to a persistent B cell lymphopenia following cerebral malaria. Given that antibody is key to development of natural immunity to malaria, this relative lack of B cells could predispose the affected children to reinfection with malaria. Perturbations of T cell subsets in severe malarial anemia and uncomplicated malaria resulted in increased proportions of children with reversed CD4/CD8 ratios (ratio of <1.0). Such a result has been proposed as a surrogate for HIV infection in African children (26). Therefore, the CD4/CD8 ratio should not be used in this way in the presence of active clinical infections, particularly malaria.

**FIG 5:**
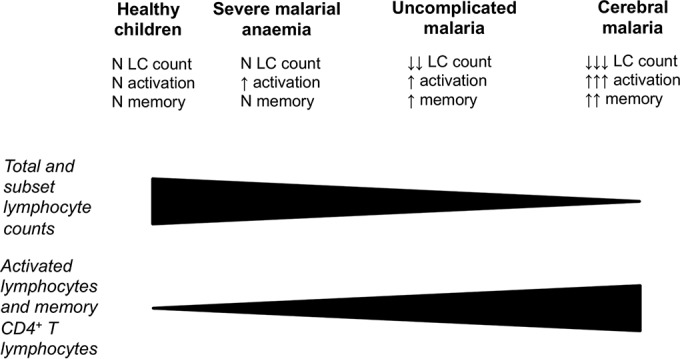
Immunologic profiles of severe malaria. Children with cerebral malaria have panlymphopenia with increased numbers of activated lymphocytes and CD4^+^ T cells with a memory phenotype. Children with severe malarial anemia have normal total and subset lymphocyte counts and similar proportions of memory CD4^+^ T cells compared with healthy age-matched children. Those with uncomplicated malaria occupy an intermediate position between these extremes. N, normal; LC, lymphocyte.

Changes in lymphocyte subsets in uncomplicated malaria reflect those in cerebral malaria, albeit to a lesser degree and with a smaller transient panlymphopenia. This is consistent with uncomplicated malaria representing an intermediate immunologic manifestation between the polarized extremes of severe malarial anemia and cerebral malaria (Fig. 5). Median lymphocyte subset values in children with severe malarial anemia are very similar to those of control children, but with a wider distribution of values, particularly for absolute lymphocyte subset counts. This could reflect a state of immunological naivety and/or unresponsiveness, particularly given the predominance of severe malarial anemia among younger children, resulting in the development of severe disease in this form of malaria. Such children may be best protected through immunization when licensed vaccines against malaria become available. Lymphocyte activation demonstrated by expression of CD69 is characteristic of all three clinical forms of malaria. This is most apparent with cerebral malaria, and excessive lymphocyte activation could contribute to pathogenesis. What is unexpected is the high proportion of CD69-expressing NK and γδ T lymphocytes. If the immunopathology of cerebral malaria results from priming of T lymphocytes by previous exposure to malaria (9), activation would be anticipated most in the adaptive CD4^+^ and CD8^+^ lymphocyte populations. A possible explanation is that malaria immunopathology is driven by innate lymphocytes and that cerebral malaria results when this arm of the immune response is triggered through inherent susceptibility to overrespond or through priming. Both NK cells and γδ T cells have been implicated in the production of proinflammatory cytokines gamma interferon (IFN-γ) and tumor necrosis factor alpha (TNF-α) in malaria (27–29).

The increased activation of lymphocytes in cerebral malaria provides a potential clue concerning the correct explanation for malaria panlymphopenia. CD69 interacts with sphingosine 1-phosphate receptor 1 in secondary lymphoid tissue, leading to the halting of lymphocyte transit, preventing their return to the circulation (30). If this occurs in malaria, the reduction in lymphocyte surface CD69 expression in convalescence would enable the rapid restoration of circulating lymphocyte levels observed posttherapy (13). This explanation for the panlymphopenia could be further investigated by the examination of secondary lymphoid tissues in postmortem studies of children with different forms of malaria. A need for T cell priming in the pathogenesis of cerebral malaria is supported by increased memory to naive CD4^+^ lymphocytes compared with control children. No such increase is seen among CD8^+^ T cell and B cell subsets. Consistent with the immunologic polarization concept for different clinical forms of malaria, a smaller increase in memory CD4^+^ T cells is found among children with uncomplicated malaria, while the median ratio for severe malarial anemia is the same as that for controls.

There are limitations to the current study. While lymphocyte subsets were enumerated and characterized, the antigen specificity of T and B cells for malaria and other pathogens was not investigated. Children who were febrile due to causes other than malaria were not included, so we are unable to compare our findings of changes in lymphocyte subsets with those in other febrile illnesses. Further studies are required to address these points.

The immunologic polarization of these different forms of clinical malaria raises a key question concerning cause and effect. Are some African children predisposed toward developing cerebral malaria and others to severe malarial anemia, while the majority suffer from only uncomplicated malaria, and what is the role of prior immunologic priming? To understand this will require further studies but has potential implications for clinical management, particularly of children with the severe forms of malaria. If children who develop severe malarial anemia represent a state of prior immune naivety, such children may stand to benefit the most from vaccination against malaria. Meanwhile, children who develop cerebral malaria may have a combination of prior immunological priming to malaria and an underlying disposition to immunopathogenesis. Such children may require other clinical interventions, particularly when their malaria is refractory to standard therapy. Caution is required here since a trial in the Gambia of monoclonal antibody to TNF-α for children with cerebral malaria resulted in no improvement in survival and an increase in neurological sequelae (31). The challenge of applying these findings to vaccine development is more difficult. Since vaccines are given to healthy children, it is not clear at the time of vaccination whether a child has an inherent predisposition toward cerebral malaria or severe malaria anemia. Studies following children in malarious regions longitudinally will help us to better understand which children are susceptible to developing either form of severe malaria and provide further scope for lymphocyte subset evaluation with time in individual children both in health and in disease.

## Supplementary Material

Supplemental material

## References

[1] World Health Organization. 2011 Global Malaria Programme, World Health Organization world malaria report 2011. World Health Organization, Geneva, Switzerland http://www.who.int/malaria/world_malaria_report_2011/en/ Accessed 29 September 2014.

[2] RTS, S Clinical Trials Partnership. 2015 Efficacy and safety of RTS, S/AS01 malaria vaccine with or without a booster dose in infants and children in Africa: final results of a phase 3, individually randomised, controlled trial. Lancet 386:31–45. doi:10.1016/S0140-6736(15)60721-8. 25913272 PMC5626001

[3] Greenwood BM, Fidock DA, Kyle DE, Kappe SH, Alonso PL, Collins FH, Duffy PE 2008 Malaria: progress, perils, and prospects for eradication. J Clin Invest 118:1266–1276. doi:10.1172/JCI33996. 18382739 PMC2276780

[4] Engwerda CR, Good MF 2005 Interactions between malaria parasites and the host immune system. Curr Opin Immunol 17:381–387. doi:10.1016/j.coi.2005.05.010. 15950450

[5] Gardner MJ, Hall N, Fung E, White O, Berriman M, Hyman RW, Carlton JM, Pain A, Nelson KE, Bowman S, Paulsen IT, James K, Eisen JA, Rutherford K, Salzberg SL, Craig A, Kyes S, Chan MS, Nene V, Shallom SJ, Suh B, Peterson J, Angiuoli S, Pertea M, Allen J, Selengut J, Haft D, Mather MW, Vaidya AB, Martin DM, Fairlamb AH, Fraunholz MJ, Roos DS, Ralph SA, McFadden GI, Cummings LM, Subramanian GM, Mungall C, Venter JC, Carucci DJ, Hoffman SL, Newbold C, Davis RW, Fraser CM, Barrell B 2002 Genome sequence of the human malaria parasite Plasmodium falciparum. Nature 419:498–511. doi:10.1038/nature01097. 12368864 PMC3836256

[6] Winzeler EA 2008 Malaria research in the post-genomic era. Nature 455:751–756. doi:10.1038/nature07361. 18843360 PMC2705782

[7] Langhorne J, Ndungu FM, Sponaas AM, Marsh K 2008 Immunity to malaria: more questions than answers. Nat Immunol 9:725–732. doi:10.1038/ni.f.205. 18563083

[8] Bull PC, Lowe BS, Kortok M, Molyneux CS, Newbold CI, Marsh K 1998 Parasite antigens on the infected red cell surface are targets for naturally acquired immunity to malaria. Nat Med 4:358–360. doi:10.1038/nm0398-358. 9500614 PMC3836255

[9] Riley EM 1999 Is T-cell priming required for initiation of pathology in malaria infections? Immunol Today 20:228–233. doi:10.1016/S0167-5699(99)01456-5. 10322302

[10] Schofield L, Grau GE 2005 Immunological processes in malaria pathogenesis. Nat Rev Immunol 5:722–735. doi:10.1038/nri1686. 16138104

[11] Chougnet C, Tallet S, Ringwald P, Deloron P 1992 Kinetics of lymphocyte subsets from peripheral blood during a Plasmodium falciparum malaria attack. Clin Exp Immunol 90:405–408. 1458676 10.1111/j.1365-2249.1992.tb05859.x PMC1554569

[12] Worku S, Bjorkman A, Troye-Blomberg M, Jemaneh L, Farnert A, Christensson B 1997 Lymphocyte activation and subset redistribution in the peripheral blood in acute malaria illness: distinct gammadelta+ T cell patterns in Plasmodium falciparum and P. vivax infections. Clin Exp Immunol 108:34–41. doi:10.1046/j.1365-2249.1997.d01-981.x. 9097908 PMC1904634

[13] Hviid L, Kurtzhals JA, Goka BQ, Oliver-Commey JO, Nkrumah FK, Theander TG 1997 Rapid reemergence of T cells into peripheral circulation following treatment of severe and uncomplicated Plasmodium falciparum malaria. Infect Immun 65:4090–4093. 9317012 10.1128/iai.65.10.4090-4093.1997 PMC175588

[14] Kassa D, Petros B, Mesele T, Hailu E, Wolday D 2006 Characterization of peripheral blood lymphocyte subsets in patients with acute Plasmodium falciparum and P. vivax malaria infections at Wonji Sugar Estate, Ethiopia. Clin Vaccine Immunol 13:376–379. doi:10.1128/CVI.13.3.376-379.2006. 16522780 PMC1391951

[15] Korir JC, Magambo JK, Mwatha JK, Waitumbi JN 2012 B-cell activity in children with malaria. Malar J 11:66. doi:10.1186/1475-2875-11-66. 22405566 PMC3325160

[16] Gupta S, Hill AV, Kwiatkowski D, Greenwood AM, Greenwood BM, Day KP 1994 Parasite virulence and disease patterns in Plasmodium falciparum malaria. Proc Natl Acad Sci U S A 91:3715–3719. doi:10.1073/pnas.91.9.3715. 8170975 PMC43652

[17] Mandala WL, MacLennan JM, Gondwe EN, Ward SA, Molyneux ME, MacLennan CA 2010 Lymphocyte subsets in healthy Malawians: implications for immunologic assessment of HIV infection in Africa. J Allergy Clin Immunol 125:203–208. doi:10.1016/j.jaci.2009.10.010. 19944455 PMC2887487

[18] Jones DS, Abrams E, Ou CY, Nesheim S, Connor E, Davenny K, Thomas P, Sawyer M, Krasinski K, Bamji M 1993 Lack of detectable human immunodeficiency virus infection in antibody-negative children born to human immunodeficiency virus-infected mothers. Pediatr Infect Dis J 12:222–227. doi:10.1097/00006454-199303000-00010. 8451099

[19] Wyler DJ 1976 Peripheral lymphocyte subpopulations in human falciparum malaria. Clin Exp Immunol 23:471–476. 780013 PMC1538392

[20] Greenwood BM, Oduloju AJ, Stratton D 1977 Lymphocyte changes in acute malaria. Trans R Soc Trop Med Hyg 71:408–410. doi:10.1016/0035-9203(77)90039-6. 304268

[21] Maina RN, Walsh D, Gaddy C, Hongo G, Waitumbi J, Otieno L, Jones D, Ogutu BR 2010 Impact of Plasmodium falciparum infection on haematological parameters in children living in Western Kenya. Malar J 9(Suppl 3):S4. doi:10.1186/1475-2875-9-S3-S4. 21144084 PMC3002140

[22] Olliaro P, Djimde A, Dorsey G, Karema C, Mårtensson A, Ndiaye JL, Sirima SB, Vaillant M, Zwang J 2011 Hematologic parameters in pediatric uncomplicated Plasmodium falciparum malaria in sub-Saharan Africa. Am J Trop Med Hyg 85:619–625. doi:10.4269/ajtmh.2011.11-0154. 21976561 PMC3183766

[23] Hviid L, Kemp K 2000 What is the cause of lymphopenia in malaria? Infect Immun 68:6087–6089. doi:10.1128/IAI.68.10.6087-6089.2000. 11203040 PMC101581

[24] Kern P, Dietrich M, Hemmer C, Wellinghausen N 2000 Increased levels of soluble Fas ligand in serum in Plasmodium falciparum malaria. Infect Immun 68:3061–3063. doi:10.1128/IAI.68.5.3061-3063.2000. 10769016 PMC97531

[25] Matsumoto J, Kawai S, Terao K, Kirinoki M, Yasutomi Y, Aikawa M, Matsuda H 2000 Malaria infection induces rapid elevation of the soluble Fas ligand level in serum and subsequent T lymphocytopenia: possible factors responsible for the differences in susceptibility of two species of *Macaca* monkeys to Plasmodium coatneyi infection. Infect Immun 68:1183–1188. doi:10.1128/IAI.68.3.1183-1188.2000. 10678924 PMC97265

[26] Zijenah LS, Katzenstein DA, Nathoo KJ, Rusakaniko S, Tobaiwa O, Gwanzura C, Bikoue A, Nhembe M, Matibe P, Janossy G 2005 T lymphocytes among HIV-infected and -uninfected infants: CD4/CD8 ratio as a potential tool in diagnosis of infection in infants under the age of 2 years. J Transl Med 3:6. doi:10.1186/1479-5876-3-6. 15683549 PMC549040

[27] D'Ombrain MC, Hansen DS, Simpson KM, Schofield L 2007 Gammadelta-T cells expressing NK receptors predominate over NK cells and conventional T cells in the innate IFN-gamma response to Plasmodium falciparum malaria. Eur J Immunol 37:1864–1873. doi:10.1002/eji.200636889. 17557374

[28] Horowitz A, Newman KC, Evans JH, Korbel DS, Davis DM, Riley EM 2010 Cross-talk between T cells and NK cells generates rapid effector responses to Plasmodium falciparum-infected erythrocytes. J Immunol 184:6043–6052. doi:10.4049/jimmunol.1000106. 20427769

[29] Stanisic DI, Cutts J, Eriksson E, Fowkes FJI, Rosanas-Urgell A, Siba P, Laman M, Davis TME, Manning L, Mueller I, Schofield L 2014 γδ T cells and CD14+ monocytes are predominant cellular sources of cytokines and chemokines associated with severe malaria. J Infect Dis 210:295–305. doi:10.1093/infdis/jiu083. 24523513

[30] Shiow LR, Rosen DB, Brdickova N, Xu Y, An J, Lanier LL, Cyster JG, Matloubian M 2006 CD69 acts downstream of interferon-alpha/beta to inhibit S1P1 and lymphocyte egress from lymphoid organs. Nature 440:540–544. doi:10.1038/nature04606. 16525420

[31] Van Hensbroek MB, Palmer A, Onyiorah E, Schneider G, Jaffar S, Dolan G, Memming H, Frenkel J, Enwere G, Bennett S, Kwiatkowski D, Greenwood B 1996 The effect of a monoclonal antibody to tumor necrosis factor on survival from childhood cerebral malaria. J Infect Dis 174:1091–1097. doi:10.1093/infdis/174.5.1091. 8896514

