# Supplementary material for: Lymphocyte Perturbations in Malawian Children with Severe and Uncomplicated Malaria
Source: Clin Vaccine Immunol. 2016 Feb 5;23(2):95–103. doi: 10.1128/CVI.00564-15 (PMC4744922; doi:10.1128/CVI.00564-15)
Supplement: Supplemental material [file supp_23_2_95__index.html]

Lymphocyte Perturbations in Malawian Children with Severe and Uncomplicated Malaria — Supplemental material 

# Lymphocyte Perturbations in Malawian Children with Severe and Uncomplicated Malaria

## Supplemental material

- Supplemental file 1 -

  Table S1. Monoclonal antibodies used and corresponding cell populations. Table S2. Monoclonal antibodies used in this study. Fig. S1. Flow cytometric gating strategy.

  PDF, 533K
