# Supplementary material for: Lymphocyte Perturbations in Malawian Children with Severe and Uncomplicated Malaria
Source: Clin Vaccine Immunol. 2016 Feb 5;23(2):95–103. doi: 10.1128/CVI.00564-15 (PMC4744922; doi:10.1128/CVI.00564-15)
Supplement: Supplemental material [file CVI.00564-15_zcd999095295so1.pdf]

Table S1. Monoclonal antibodies used and corresponding cell populations

	FITC	PE	PerCP	APC	Identity of subsets
1	moGI	moGI	moGI	moGI	Isotype control
2	-	CD27	-	CD19	Memory/naïve B cells
3	-	CD56	CD3	CD69	NK/NKT, activation status
4	TCR-γ/δ	-	-	CD69	$\gamma\delta^{+}$ T cells, activation status
5	-	CD4	CD3	CD8	$CD4^{+}$ and $CD8^{+}$ T cells
6	CD45RA	CD45RO	CD4	CD8	Memory/naïve $CD4^{+}$ and $CD8^{+}$ T cells
7	-	-	-	-	Negative control

Table S2: Monoclonal antibodies used in study

Antibody	Product Number	Supplier
Anti-CD3-PerCP	560835	BD
Anti-CD4-PE	555347	BD
Anti-CD4-PerCP	560650	BD
Anti-CD8-APC	561952	BD
Anti-CD19-APC	641395	BD
Anti-CD27-PE	340425	BD
Anti-CD45RA-FITC	555488	Pharm
Anti-CD45RO-PE	555493	Pharm
Anti-CD56-PE	555516	BD
Anti-CD69-APC	555533	BD
Anti-TCR-γ/δ-FITC	347903	BD
Anti-moGI-FITC	555909	BD
Anti-moGI-PE	555749	BD
Anti-moGI-PerCP	555751	BD
Anti-moGI- APC	550795	BD

BD = Becton Dickinson Pharm = Pharmingen.

Fluorescein isothiocyanate (FITC), Phycoerythrin (PE),

Peridinin chlorophyll protein (PerCP), and Allophycocyanin (A)

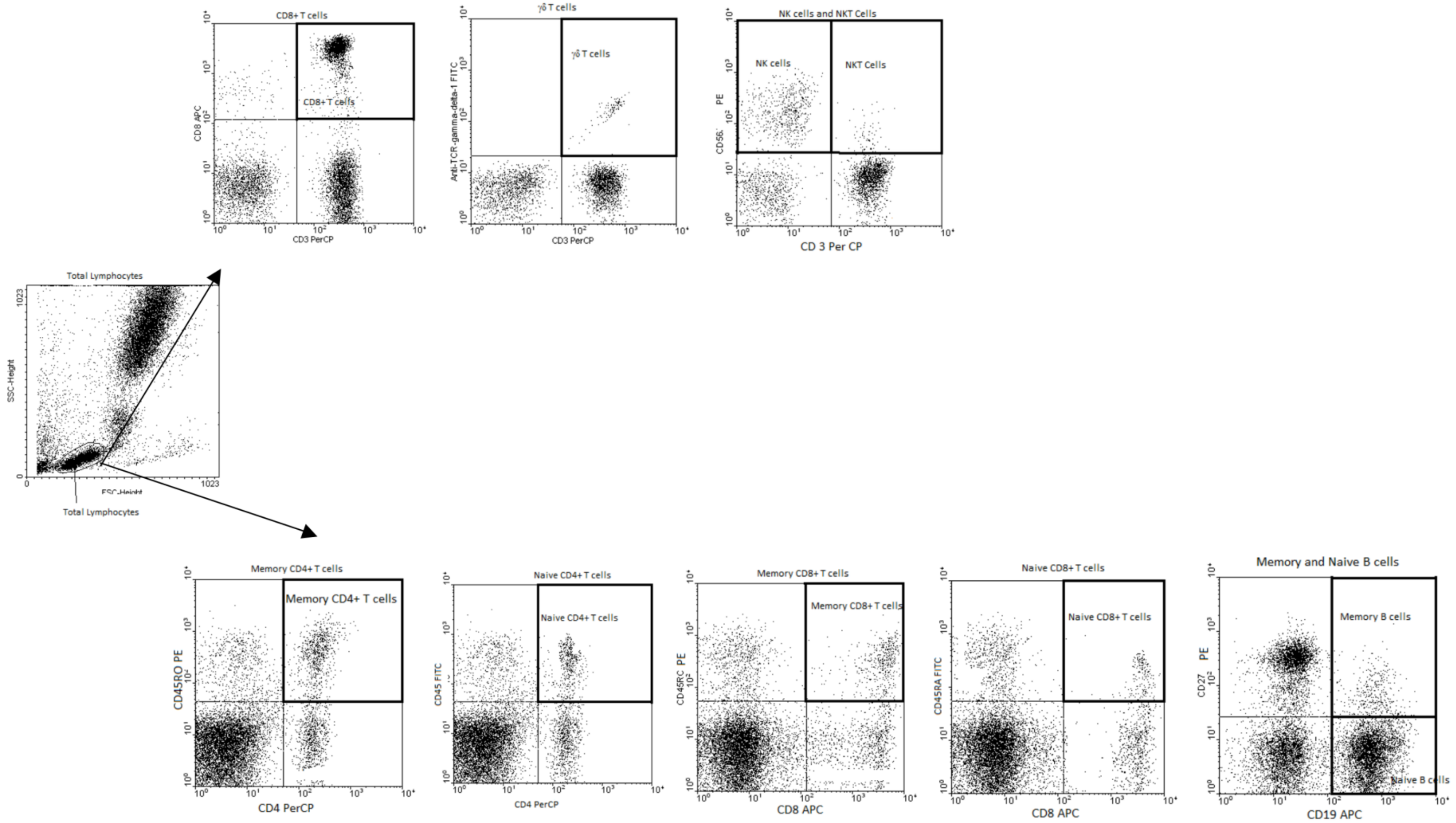

Figure S1: **Flow cytometric Gating Strategy**
